# Assessment of Climate Change Impacts on the Distribution of Endangered and Endemic *Changnienia amoena* (Orchidaceae) Using Ensemble Modeling and Gap Analysis in China

**DOI:** 10.1002/ece3.70636

**Published:** 2024-11-25

**Authors:** Ting Liu, Hanwei Cai, Guangfu Zhang

**Affiliations:** ^1^ Jiangsu Key Laboratory of Biodiversity and Biotechnology, School of Life Sciences Nanjing Normal University Nanjing China

**Keywords:** climate change, conservation, distribution range, environmental factors, Orchidaceae

## Abstract

Climate change has significant impacts on the distribution of orchids. The endemic and endangered orchids are more susceptible to climate change than widely distributed orchids. To date, little is known concerning the response of endangered *Changnienia amoena*, endemic to China, to different climate scenarios. Here, we build an ensemble model comprising random forest model, maximum entropy model, and gradient boosting model in Biomod2 package to project its potential distribution in China, evaluate its current protective effectiveness, and identify its conservation gaps in China by determining the *C. amoena* population range within the natural protected areas. The outcomes showed that the four key environmental factors influencing its distribution were mean diurnal temperature range, minimum temperature of the coldest month, temperature seasonality, and precipitation of the warmest quarter. This orchid was currently distributed mainly in southern Anhui, central and western Hubei, western Hunan, southern Shaanxi, and eastern Sichuan province. The total suitable area of *C. amoena* was 58.33 × 10^4^ km^2^, only accounting for 6.08% of China's total territory, which is larger than known. However, only 4.48% of the suitable area is located within national nature reserves and 3.33% within provincial nature reserves, respectively. During the last inter glacial and mid‐holocene, its suitable areas were larger than the current. Under six future climate scenarios, its suitable areas may decrease averagely by 2.26% relative to the current, with severe habitat fragmentation. Collectively, the centroid of *C. amoena* is expected to shift towards the southeast in the future. Therefore, our findings demonstrate that climate change has an adverse effect on its potential distribution. We recommend expanding protected areas or establishing new conservation sites for *C. amoena* in China. Furthermore, our study can help to inform the development of conservation management strategies for other endangered Chinese endemic orchids under climate change.

## Graphical abstract

The major factor limiting Changnienia amoena distribution range is temperature‐related, and its current suitable area is 58.33 × 104 km^2^, accounting for 6.08% of China. Climate change exerts an adverse effect on its potential distribution. Over 90% of the suitable habitat for *C. amoena* is in a zero‐protection status.

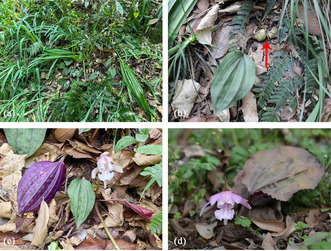

## 1 Introduction

Climate change may alter the geographical distribution of plants, especially for endemic plant species at the regional scale (Kelly and Goulden 2008; Huang, Chen, and Yu 2024). The sixth assessment report published by the Intergovernmental Panel on Climate Change (IPCC) in 2023 stated that the global surface temperature had increased by 1.1°C from 2011 to 2020 compared to the period from 1850 to 1900, and with the global warming, global temperature increase will breach the critical threshold of 1.5°C as defined by the IPCC in the near future (2021–2040) (Gao et al. 2023). With the rising temperature, there will be more pronounced changes in hydrothermal conditions than ever, which will cause corresponding shift in the potential distribution of plants (Aitken et al. 2008).

Orchidaceae is ranked as one of the richest families of seed plants worldwide. It has 814 genera and over 27,500 species and is a herbaceous taxon with many rare and endangered species (Chen et al. 2020). Orchids are widely distributed in the tropics and subtropics. Most of them have medicinal value (e.g., *Gastrodia elata* and *Dendrobium* spp.) and ornament value (e.g., *Phalaenopsis* and *Cymbidium*) (Jin, Li, and Ye 2019). Additionally, Orchidaceae is frequently considered as the flagship group for plant conservation (Hu et al. 2022). However, the species of Orchidaceae are facing a great threat because of habitat fragmentation or loss, which may be exasperated due to the ongoing global warming. Consequently, Orchidaceae has more threatened species than most other families (Swarts and Dixon 2009; Zhang, Du, et al. 2015; Zhang, Yan, et al. 2015). Hence, all orchidaceous species within this family are listed in the Appendix II of the Convention on International Trade in Endangered Species of Wild Fauna and Flora (CITES, https://cites.org/eng/app/appendices.php, accessed on 5 March 2024), accounting for over 90% of the total plant species of CITES (Jin, Li, and Ye 2019). Simultaneously, some endangered orchid species have been protected by law in many countries. For example, there are 184 orchid species listed as threatened by the Australian Government in 2018 (Wraith and Pickering 2019). There are 296 ones listed in the Chinese updated checklist, namely *the List of National Key Protected Wild Plants* issued in September of 2021, accounting for approximately 30% of the total species (https://www.forestry.gov.cn/c/www/lczc/10746.jhtml, accessed on 5 March 2024).

Indeed, endemic and endangered orchids are more susceptible to the impacts of climate change (Tsiftsis and Tsiripidis 2020). This is primarily due to their mycorrhizal specificity, pollinator specialization, and limited seed germination rates (Gravendeel et al. 2004; McCormick and Jacquemyn 2014). More importantly, such orchids usually have small population size, specific habitat requirements, and low fecundity (Jacquemyn et al. 2005; Crain and Tremblay 2014). Currently, there are few researches on the potential geographical distribution of endemic and endangered orchids. For example, Wang et al. (2015) discovered that *Spiranthes parksii* , an endangered terrestrial orchid endemic to central Texas in America, had high requirements for soil resources which were able to provide specific mycorrhizal fungi for such an orchid. They further pointed out that future climate change may make the orchid habitat more fragmented by affecting the growth and distribution of soil mycorrhiza. Boral and Moktan (2024) employed the MaxEnt model to predict the potential distribution of two endemic medicinal orchids including *Crepidium acuminatum* and *Satyrium nepalensein* in eastern Himalayan. Their findings indicate that future climate change is likely to strongly affect the two orchids' habitat suitability. Similarly, Hu et al. (2022) addressed the potential impacts of climate change on the richness and distribution of 17 endangered Orchidaceae species on the Qinghai‐Tibetan Plateau using the MaxEnt model. However, these studies only used single model (i.e., MaxEnt modeling) for distribution predictions and rarely combined the predicted areas of orchids with protected areas to analyze their conservation status.

**FIGURE 1:**
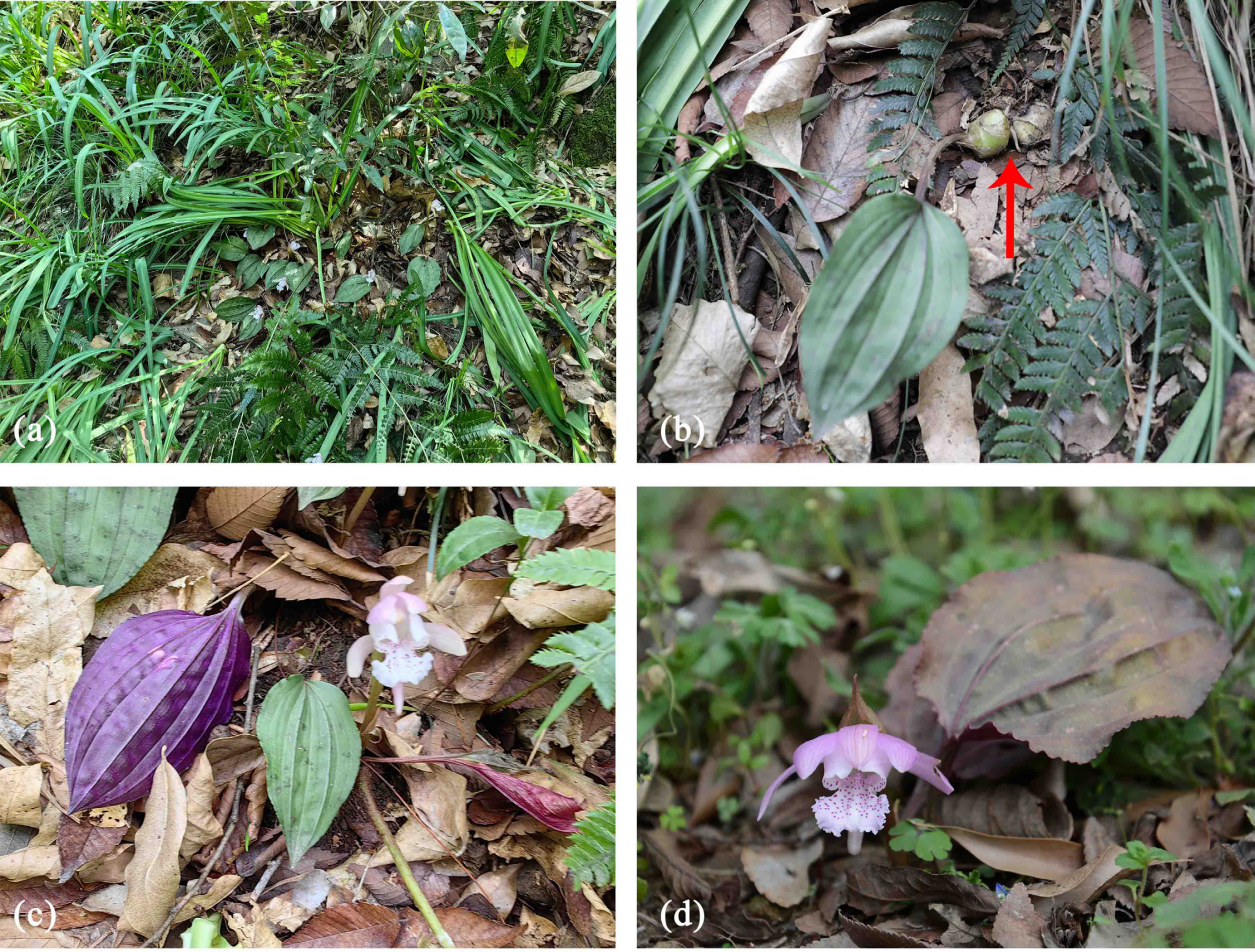
Photos of *Changnienia amoena*. (a) *C. amoena* community habitat preferences; (b) Subellipsoid to broadly ovoid pseudobulbs (red arrow); (c) A single leaf at apex of pseudobulb, abaxially purplish red (left), adaxially dark green (right); (d) A solitary inflorescence usually with a pink or white flower. The photos were taken by Guangfu Zhang.

There are 1582 species from the Orchidaceae family in China (Liu et al. 2020). Among them, 531 orchid species are endemic to China. According to the International Union for Conservation of Nature (IUCN) Red List criteria, 653 of Chinese orchid species are threatened (i.e., critically endangered, endangered, or vulnerable), accounting for 41.28% of the whole Chinese orchids (Liu et al. 2020). More than one third of these threatened orchids are listed as national‐level protected species. A typical example is *Changnienia amoena* S. S. Chien. This orchid belongs to the genus *Changnienia* in the Orchidaceae family, which is a monotypic genus endemic to China (Wu and Raven 2009). It is a perennial terrestrial herb and often grows in the understory of broad‐leaved or needle and broad‐leaved mixed forests in the mountains of eastern and central China (Figure 1a). This orchid has an elliptical or broadly ovoid fleshy pseudobulb with two or three nodes (Figure 1b). *C. amoena* has a solitary leaf at the apex of its pseudobulb, and its blade is spreading, recurved, adaxially dark green, and abaxially purplish red. Its leaf is broadly ovate to broadly elliptic (Figure 1c). This orchid usually has a solitary inflorescence, which produces only a spreading and large flower, white or pink, with purplish red spots in white lip (Figure 1d). In general, it begins to bloom from April to May and bears long ellipsoid capsules from October to November. It has high ornamental value because of its unique flower shape and bright color. Second, the whole plant or its pseudobulbs can be utilized as an important Chinese herb medicine. In addition, it can be used as an important material for systematic evolution of Orchidaceae as it is considered as a primitive taxon (Li and Ding 2021).

*C. amoena* presents small populations in distribution, most of which are scattered and discontinuous in China (Li and Ge 2006). Li et al. (2002) reported that as a self‐compatible outcrosser, *C. amoena* had low genetic diversity detected by RAPD technique. Due to its few flowers with defective floral structure, *C. amoena* fails to provide rewards for pollinators. Such a deceptive pollination strategy results in a very low fruit set rate in the field (Sun et al. 2006). We thereby speculate that this orchid heavily depends on asexual reproduction, thus making it difficult to regenerate its wild populations. Due to its low reproductive efficiency, in tandem with global warming and over‐exploitation by humans, *C. amoena* is gradually decreasing in population size and increasingly shrinking in distribution area (Sun, Luo, and Ge 2003; Li and Ge 2006). Consequently, as early as 1992, *C. amoena* was listed as a rare and endangered species in the *China Plant Red Data Book‐Rare and Endangered Plants* (Vol. I) (Fu 1992). It was classified as a second‐grade species in *the List of National Key Protected Wild Plants* in 2021. Furthermore, it has been listed as “Endangered” (EN) species in the IUCN Red List.

According to the *Flora of China*, *C. amoena* was found in Anhui, Hubei, Hunan, Jiangsu, Jiangxi, Shaanxi, Sichuan, and Zhejiang provinces in China (Wu and Raven 2009), primarily concentrated in central Hubei and Hunan provinces. In recent years, Zhang (1996) discovered *C. amoena* at a moist forest edge in Wen County, Gansu Province. Chen, Ma, and Wang (2013) found two individuals of *C. amoena* in a subtropical forest at Shuanghe Village, Chengkou County, Chongqing City, southwest China. Qin, Zou, and Meng (2018) identified a wild population of *C. amoena* in a bamboo forest with an altitude of 690 m at Mao'er Mountain National Nature Reserve, Guangxi Province. According to recent investigations and related reports, we think that *C. amoena* occurs in more than 13 provinces in China, including Anhui, Chongqing, Gansu, Guangxi, Guizhou, Henan, Hubei, Hunan, Jiangsu, Jiangxi, Shaanxi, Sichuan, and Zhejiang provinces. Therefore, it remains unclear concerning its actual distribution range in China.

Species distribution models (SDMs) are powerful tools for examining the relationships between species' potential habitats and environmental factors (Fleishman et al. 2002). SDMs primarily rely on the geographical distribution information of species and environmental data to estimate their distribution (Elith and Leathwick 2009). In view of modeling algorithm as the most important source of uncertainty in performance from SDMs, it is generally recognized that the integration of multiple algorithms may provide more accurate predictions (Watling et al. 2015). The Biomod2 model is currently a reliable multi‐model ensemble platform that utilizes different types of statistical methods to improve the predictive accuracy of the single model and increase the reliability of projecting results (Thuiller, Araújo, and Lavorel 2003; Fang et al. 2021). Each single model has its own advantages and disadvantages. Therefore, it seems a good choice to develop an ensemble model by combining different individual models. Such an ensemble model usually performs much better than single one in terms of accuracy (Araújo and New 2007). The Biomod2 package includes ten species distribution models. Namely, they are maximum entropy model (MaxEnt), Generalized linear model (GLM), generalized additive model (GAM), multiple adaptive regression spline (MARS), surface range envelope (SRE), flexible discriminant analysis (FDA), categorical regression tree analysis (CTA), gradient boosting model (GBM), random forest model (RF), and artificial neural network (ANN).

Recently, the Biomod2 model has been employed to predict the habitat suitability of endangered orchids (Dormann et al. 2018). For instance, Yu et al. (2020) used the Biomod2 to analyze the impact of climate change on the suitable habitats of both *Calanthe sieboldii* and its three pollinators in China, indicating that its distribution may be affected by future climate change and the distribution reduction of these pollinators as well. To date, there has been only one research on the potential suitable distribution of *C. amoena* , in which a single model (i.e., MaxEnt) was employed to forecast the population distribution of *C. amoena* . Unfortunately, this study only used the data of one province, that is, Jiangxi Province, eastern China (Chen 2019). In fact, *C. amoena* is distributed in more than ten provinces of China. It seems unlikely to predict its geographical distribution based on data from only one province. Therefore, it remains unclear about the potential geographical distribution of *C. amoena* in China.

Here, we first build an ensemble model generated by Biomod2 to link species distribution records of *C. amoena* with environmental variables (climate, terrain, and soil). In conjunction with ArcGIS spatial analysis, we then determine the suitable area of *C. amoena* in China under different climatic scenarios. Specifically, the objectives of this study are (1) to identify the key environmental factors influencing the distribution of *C. amoena* ; (2) to forecast the current potential suitable habitats for *C. amoena* in China; (3) to predict its suitable habitats under past and future climate scenarios, and the magnitude and direction of centroid change; and (4) to analyze the conservation gap of *C. amoena* based on the distribution data of nature reserves in China. This study will provide scientific references for the conservation management for *C. amoena* .

## 2 Materials and Methods

### 2.1 Species Occurrence Data

In our study, we collected occurrence data of *C. amoena* mainly from field survey, related websites, and published literatures. First, we obtained its presence points based on our field investigation for *C. amoena* wild populations in Anhui, Hubei, Jiangsu, Jiangxi, and Zhejiang provinces in eastern and central China during the period 2021–2023. For example, in the mid‐April of 2023, we found a wild population of *C. amoena* near a creek in a subtropical mountainous area, which is situated in Longtan, Liyang City, Jiangsu Province, eastern China.

Second, we searched, collected, and compiled original specimen records containing latitude and longitude or detailed locality information through the Chinese National Specimen Information Infrastructure (NSII, http://www.nsii.org.cn, last accessed on 16 January, 2024) and the Global Biodiversity Information Facility (GBIF, https://www.gbif.org/, last accessed on 16 January, 2024). We simultaneously searched for species name or Latin name in the Plant Photo Bank of China (PPBC, http://ppbc.iplant.cn, last accessed on 16 January, 2024) obtained detailing the locality information of the images through image library and then converted into latitude and longitude.

Third, we used both the species name and Latin name of *C. amoena* as keywords for searching the related literature, which included the *Flora of China*, local floras, published papers, and investigation reports. For a small amount of data with detailed collection locations but without corresponding coordinates, we searched on Google Earth or Baidu Maps to obtain the corresponding latitude and longitude coordinates and refined these data to two decimal places. Next, we removed duplicates, artificially introduced cultivars (such as botanical gardens), and specimen records lacking latitude and longitude information or with unidentifiable fonts.

**FIGURE 2:**
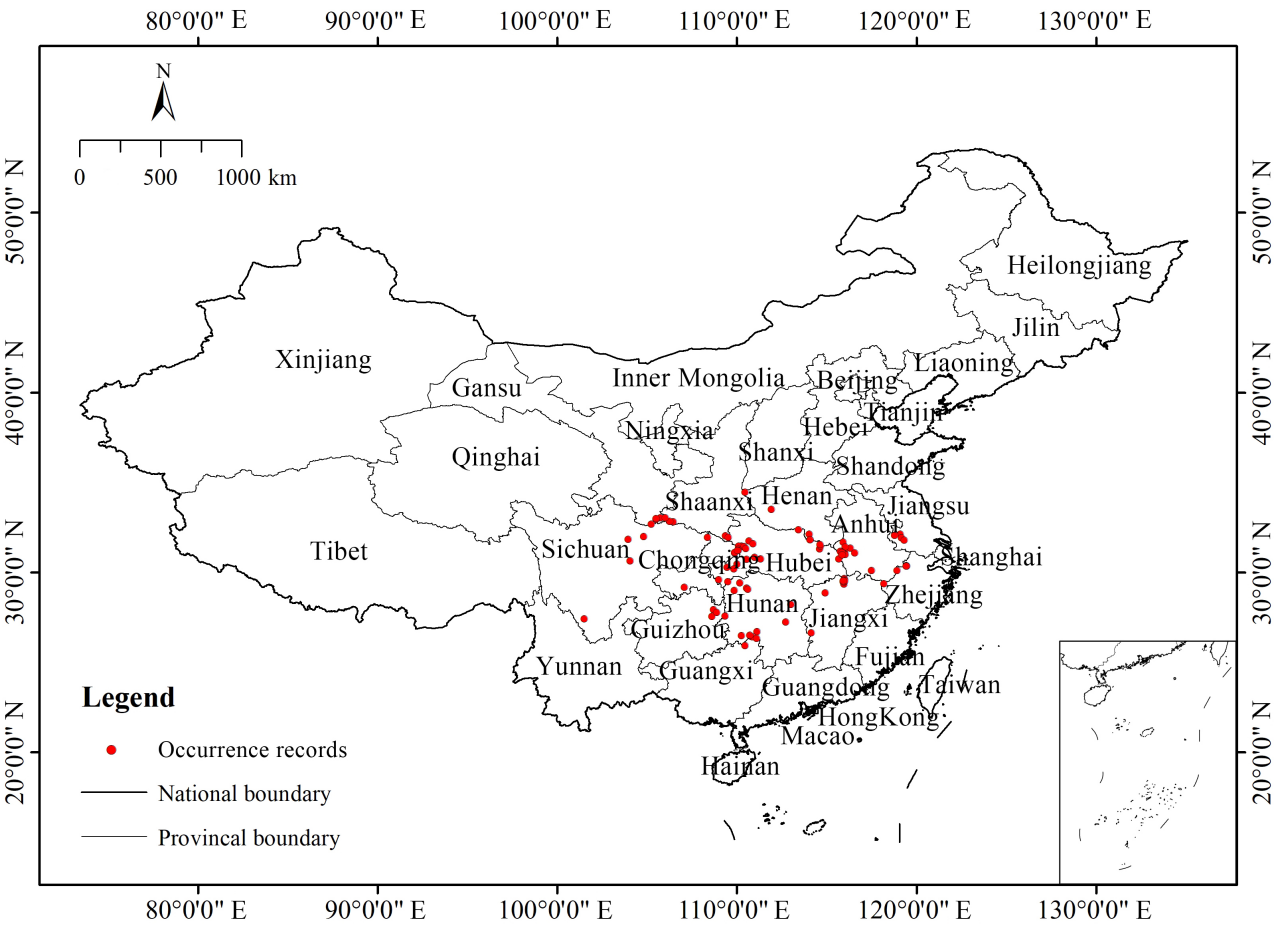
Current occurrence records of *Changnienia amoena* in China.

By doing so, we obtained 108 distribution point data of *C. amoena* . We set the resolution to rarefy data to 1 km using the spatially rarefy occurrence data for SDM tool in SDMtoolbox 2.0 to reduce sampling bias and decrease spatial autocorrelation (Brown 2014; Kong, Li, and Zou 2019). This process eliminated duplicate, erroneous, and ambiguous records of *C. amoena* within a 1 km × 1 km spatial range of the selected points. Finally, we retained 93 valid distribution records of *C. amoena* and saved these records in “.csv” format. The collected latitude and longitude of *C. amoena* distribution points are shown in Table S1, and the distribution of *C. amoena* in China is shown in Figure 2.

### 2.2 Environmental Variables

According to our field investigation and related literature analysis, we noticed that this orchid mainly occurred in Eastern and Southeastern China, especially in the subtropical mountains of China. Therefore, we selected three types of environmental data. First, the topographic data involved elevation data downloaded from the WorldClim v2.1 database (https://www.worldclim.org, last accessed on 16 January, 2024) as well as slope data obtained from the national DEM elevation data (http://www.tuxingis.com, last accessed on 16 January, 2024).

Second, the 19 bioclimatic variables (Bio1‐Bio19) were downloaded from the World Climate Database, including past, current, and future climate data. The WorldClim 1.4 dataset (Hijmans et al. 2005), based on the Coupled Model Inter‐comparison Project Phase 5 (CMIP5), was selected for paleoclimatic data, including the last interglacial (LIG; 120,000–140,000 years ago) and the mid‐holocene (MH; about 6000 years ago). Global climate models (GCMs) used for the past period were derived from the community climate system model version 4 (CCSM4), developed by the National Center for Atmospheric Research (NCAR). Current (the average between 1970 and 2000) and future (2050s and 2070s) climate data were derived from calculating the equally‐weighted average values of three global climate models: the CCSM4, the Beijing climate center climate system model version 1.1 (BCC‐CSM1‐1), and an earth system model based on the model for interdisciplinary research on climate (MIROC‐ESM) (Fick and Hijmans 2017). The representative concentration pathways (RCPs) consist of a series of greenhouse gas concentration scenarios that have been widely used to determine species' responses to climate change (Zhang et al. 2021). The three typical concentration pathways selected for this study represent different climate change scenarios ranging from the lowest to the highest emission scenario, including RCP 2.6 (representing the lowest emission scenario), RCP 4.5 (indicating a medium and stable emission scenario), and RCP 8.5 (representing the highest emission scenario). Each pathway includes two periods (i.e., 2050s and 2070s), using the average emissions for the years 2041–2060 and 2061–2080, respectively.

Third, 17 types of topsoil (0–30 cm) data were downloaded from the National Tibetan Plateau Scientific Data Center based on the Harmonized World Soil Database v1.2 (HWSD, http://www.tpdc.ac.cn/zh‐hans/). The environmental data were ultimately saved in ASCII format. The unified spatial resolution of the data is 30 arc sec. Additionally, excessive environmental variables can increase the dimensionality of ecological space, which can lead to over‐fitting or inaccurate modeling. We thereby performed a Pearson correlation coefficient test for reducing multicollinearity among environmental factors. For two environmental variables with a correlation coefficient |*r*| > 0.8, the larger contribution one was retained (Dormann et al. 2013; Jiang et al. 2018).

Ultimately, after screening, Table 1 shows the environmental variables of subsequent modeling in different periods and their corresponding contribution rates.

**TABLE 1:** Description of environmental variables and percent contribution of variables (in bold font) used in the final the ensemble model under different climate scenarios.

Environmental variables	Description	Unit	Percent contribution (%)		
			LIG	MH	Current
Bio2	Mean diurnal range (mean of monthly (max temp–min temp))	°C	**38.6**	**37.8**	**43.7**
Bio3	Isothermality ((Bio2/Bio7) × 100)	%	**6.6**	**2.4**	—
Bio4	Temperature seasonality (standard deviation × 100)	—	—	**7.1**	**14.4**
Bio5	Max temperature of warmest month	°C	**0.4**	—	—
Bio6	Min temperature of coldest month	°C	—	—	**14.8**
Bio7	Temperature annual range (Bio5–Bio6)	°C	**16.2**	—	—
Bio8	Mean temperature of wettest quarter	°C	—	**0.1**	**0.4**
Bio11	Mean temperature of coldest quarter	°C	**9.4**	—	—
Bio12	Annual precipitation	mm	—	**6.4**	—
Bio13	Precipitation of wettest month	mm	**0.1**	**2.4**	—
Bio14	Precipitation of driest month	mm	—	—	**0.6**
Bio15	Precipitation seasonality (coefficient of variation)	—	**0.4**	**12**	—
Bio18	Precipitation of warmest quarter	mm	—	**3.7**	**13.2**
Bio19	Precipitation of coldest quarter	mm	**1.3**	—	—
Elevation	—	m	—	—	**1.6**
Slope	—	°	—	—	**2.6**
T‐BS	Topsoil Base Saturation	%	—	—	**0.3**
T‐CaCO_3_	Topsoil Calcium Carbonate	%	—	—	**0.3**
T‐CEC‐CLAY	Topsoil CEC (clay)	—	—	—	**0.7**
T‐CLAY	Topsoil Clay Fraction	%	—	—	**0.6**
T‐ESP	Topsoil Sodicity	—	—	—	**0.6**
T‐GRAVEL	Topsoil Gravel Content	%	—	—	**0.2**
T‐OC	Topsoil Organic Carbon	%	—	—	**1.9**
T‐SILT	Topsoil Silt Fraction	%	—	—	**0.1**
T‐TEB	Topsoil Exchangeable Base	—	—	—	**0.1**

*Note:* LIG and MH mean the last interglacial and the mid‐holocene, respectively.

### 2.3 Species Distribution Modeling Methodology

First, to evaluate the performance of SDMs, based on the occurrence data and environmental variables of *C. amoena* , we modeled the potential geographical distribution of *C. amoena* under climate change using the ten different model algorithms in the Biomod2 package. During the modeling process, R4.3.3 randomly generated 1000 pseudo‐presence points. 75% of the distribution points were randomly selected for model training, with the remaining 25% used to assess the accuracy of the model predictions. Moreover, to ensure the predictive accuracy of the models, this operation was repeated ten times to obtain the average value as the final modeling result, yielding the area under curve (AUC) and true skill statistics (TSS) values of each model. We used AUC and TSS to evaluate model performance because the combination of the two values can improve the reliability of model evaluation (Liu, White, and Newell 2013; Wang et al. 2019). The value of AUC ranges from 0 to 1. For each model, the larger the AUC value, the stronger the correlation between the model and the environmental variables and accordingly the higher the accuracy of its prediction outcome (Pavlović et al. 2019). The AUC value is classified into five levels: (1) Excellent: 0.90–1.00. (2) Good: 0.80–0.90. (3) Fair: 0.70–0.80. (4) Poor: 0.60–0.70. (5) Failure: 0.50–0.60 (Phillips and Dudík 2008; Jalaeian et al. 2018). The TSS is based on a method improved from Kappa, and it also takes into account the maximum specificity and sensitivity threshold. It not only retains the advantages of Kappa but also corrects the drawbacks of Kappa's susceptibility to the extent of species distribution (Allouche, Tsoar, and Kadmon 2006). The TSS index is calculated as TSS = Sensitivity + Specificity − 1. Generally, the TSS value ranges from −1 to 1. If the TSS value is greater than 0.8, this indicates a good model. A value of 0.5 or less reflects that the predictive performance is worse than random prediction (Allouche, Tsoar, and Kadmon 2006; Wang, Zhi, and Zhang 2024). We selected the top three models with AUC > 0.95 and TSS > 0.8 from the ten models as the excellent predictive models to form an ensemble model. Then, we compared the model performance of the mixed ensemble model and the default one in Biomod2 package. Finally, using the optimal combined model algorithm, we obtained the ensemble model results of nine climate scenarios (i.e., LIG, MH, current, and six future climate scenarios). Subsequently, these predictive results were output as maps in ArcGIS 10.8, showing the probability of the presence of *C. amoena* at each grid in the study area.

### 2.4 Geospatial Analysis

To directly display the potential range changes of *C. amoena* under different climate scenarios, we utilized ArcMap 10.8 to visualize the data generated after running the models. The reclassification of model results was based on the “test sensitivity and specificity threshold” (0.20) when only the presence data were available (Liu, White, and Newell 2013). The habitat suitability of *C. amoena* was divided into four levels: unsuitable area (0.00–0.20), low suitable area (0.20–0.46), moderately suitable area (0.46–0.73), and highly suitable area (0.73–1.00). The sum of the moderately and highly suitable area is considered the total suitable habitat (Guillera‐Arroita et al. 2015). Finally, the reclassified maps of the potential suitable area of *C. amoena* were generated in ArcMap, and the SDM toolbox v2.5 (Brown, Bennett, and French 2017; Wang, Zhi, and Zhang 2024) was employed to calculate distribution changes and centroid shifts of suitable area.

### 2.5 Conservation Gap Analysis

The dataset of nature reserves was derived from the most recent official list of the Ministry of Ecological Environment of China (http://www.mee.gov.cn, last accessed on 7 March, 2024) and the World Database on Protected Areas (http://www.protectedplanet.net/, last accessed on 7 March, 2024). After excluding marine protected areas, we developed a map layer of China's protected areas, which included 464 national and 806 provincial nature reserves, reflecting the current status of protected areas in China (Yu et al. 2023). The total protected area used in this study was 97.18 × 10^4^ km^2^, accounting for approximately 10.12% of China's total land area. The practice of plant conservation is generally based on the actual distribution of a species and its predicted geographical distribution under current climate scenario is considered to be the closest to its actual distribution. Therefore, we used ArcGIS v10.8 to overlay the identified current suitable grids for *C. amoena* with the layers of protected areas (i.e., national and provincial levels) to determine the *C. amoena* population range within the natural protected areas, to evaluate its current protective effectiveness, and to identify its conservation gaps in China (Yang et al. 2021; Xue et al. 2021). When a suitable grid of *C. amoena* falls within the Chinese natural protected area, this indicates that its population in this grid is protected; otherwise, it is considered a conservation gap (Chi et al. 2017). Then, we calculated the area of its suitable habitat within the protected areas and its corresponding proportion (i.e., the protection rate), respectively.

## 3 Results

### 3.1 Optimal Model and Model Evaluation

We performed ten individual models using Biomod2 for *C. amoena* with 93 occurrence records. The AUC and TSS values for the 10 individual models in Biomod2 were presented in Table 2 (the AUC and TSS values were described as the mean ± standard deviation (SD)). The ten models had the AUC values ranging from 0.7655 to 0.9680 and in terms of AUC, the first three models were RF, GBM, and MaxEnt, respectively. Similarly, the ten models had the TSS values ranging from 0.5311 to 0.8596, and in terms of TSS, the first three models were GBM, MaxEnt, and RF, respectively. This indicated that whether in terms of AUC or TSS, the three models were consistently ranked in the top three. However, the SRE model had the lowest values of AUC and TSS. This indicated that there were varying degrees of model reliability among the ten individual models in Biomod2 for *C. amoena* . Therefore, we chose RF, GBM, and MaxEnt to form a new ensemble model. Moreover, each of the three models had mean AUC > 0.95 and TSS > 0.8.

**TABLE 2:** Comparison of mean value (±SD) of the area under curve (AUC) and true skill statistic (TSS) of individual models and ensemble model.

Models	AUC	TSS
ANN	0.9082 ± 0.1260	0.7526 ± 0.1259
CTA	0.9098 ± 0.1259	0.8120 ± 0.1258
FDA	0.9393 ± 0.1269	0.7587 ± 0.1272
GAM	0.8127 ± 0.1202	0.6222 ± 0.1215
GBM	0.9653 ± 0.1217	0.8596 ± 0.1214
GLM	0.9301 ± 0.1214	0.8029 ± 0.1214
MARS	0.9358 ± 0.1269	0.8122 ± 0.1269
RF	0.9680 ± 0.1270	0.8430 ± 0.1267
SRE	0.7655 ± 0.1262	0.5311 ± 0.1267
MaxEnt	0.9550 ± 0.0010	0.8577 ± 0.0136
Ensemble model	0.9940 ± 0.1270	0.9160 ± 0.1269
Ensemble model (GBM, RF, and MaxEnt)	0.9950 ± 0.0056	0.9330 ± 0.0074

Such a new ensemble model generated a mean AUC of 0.9950 and TSS of 0.9330, respectively. It presented higher values of AUC and TSS than the default ensemble model of ten individual models, indicating that the former had a higher predictive accuracy. Moreover, the new ensemble model simultaneously displayed the lower SD than the default one, showing better stability. Therefore, the new ensemble model was markedly superior to the default one. Accordingly, we chose the three optimal individual models (i.e., RF, GBM, and MaxEnt) to build an ensemble model. The subsequent analysis was performed in the mixed effect model.

### 3.2 Key Environmental Variables

We calculated the contribution rates of each environmental variable using the ensemble model and selected these variables significantly affecting the distribution of *C. amoena* . The top four environmental variables with the highest contribution rates on the potential distribution of *C. amoena* were identified as the key environmental factors (Table 3). Namely, they were mean diurnal temperature range (Bio2, 43.7%), minimum temperature of the coldest month (Bio6, 14.8%), temperature seasonality (Bio4, 14.4%), and precipitation of the warmest quarter (Bio18, 13.2%). The total contribution value of temperature factors (i.e., Bio2, Bio6, Bio4) reached 72.9%. When the species existence probability was greater than 0.46, it indicated that the range of environmental variables was suitable for the growth of this orchid (Ren et al. 2020; Yan and Zhang 2022).

**TABLE 3:** Key climatic factors influencing habitat distribution of *Changnienia amoena*.

Environmental variables	Percent contribution (%)	Suitable range	Optimum	Maximum probability of existence
Bio2 (°C)	43.7	6.8 ~ 8.9	7.4	0.73
Bio6 (°C)	14.8	−6.0 ~ 2.5	−2	0.85
Bio4	14.4	7.2 ~ 9.1 (×100)	7.8 (×100)	0.82 (×100)
Bio18 (mm)	13.2	420 ~ 720	625	0.84

**FIGURE 3:**
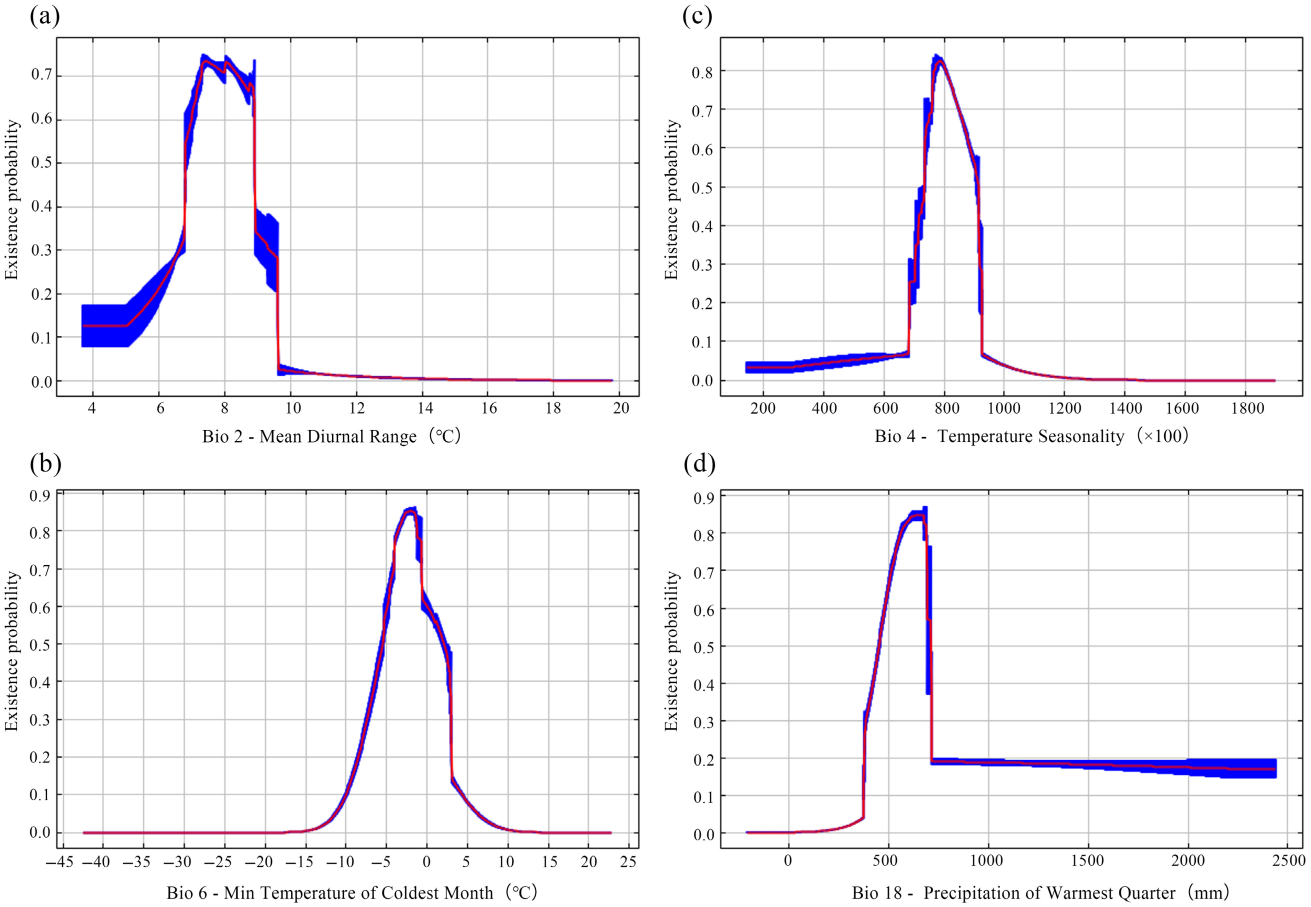
Response curves of *Changnienia amoena* to key environmental variables.

According to the response curves, the optimal range of Bio2 for *C. amoena* growth was 6.8°C–8.9°C, with the most suitable Bio2 being 7.4°C (Figure 3a). The optimal growth range of Bio6 was −6.0°C to 2.5°C, and when the temperature increased, the existence probability of *C. amoena* gradually increased, peaking at −2.0°C (0.84) (Figure 3b). When Bio4 was 720, it was suitable for the growth of *C. amoena* . The probability of its existence gradually increased as Bio4 increased, reaching the maximum at 780 (0.82). Then, the probability of existence decreased with the coefficient ranging from 780 to 910 (Figure 3c). When the precipitation of the warmest quarter (Bio18) exceeded 420 mm, the existence probability was over 0.46 and the maximum probability of existence was 0.84 at 625 mm. Within the range of 420–720 mm, the existence probability initially increased then decreased (Figure 3d).

### 3.3 Current Suitable Distribution

**FIGURE 4:**
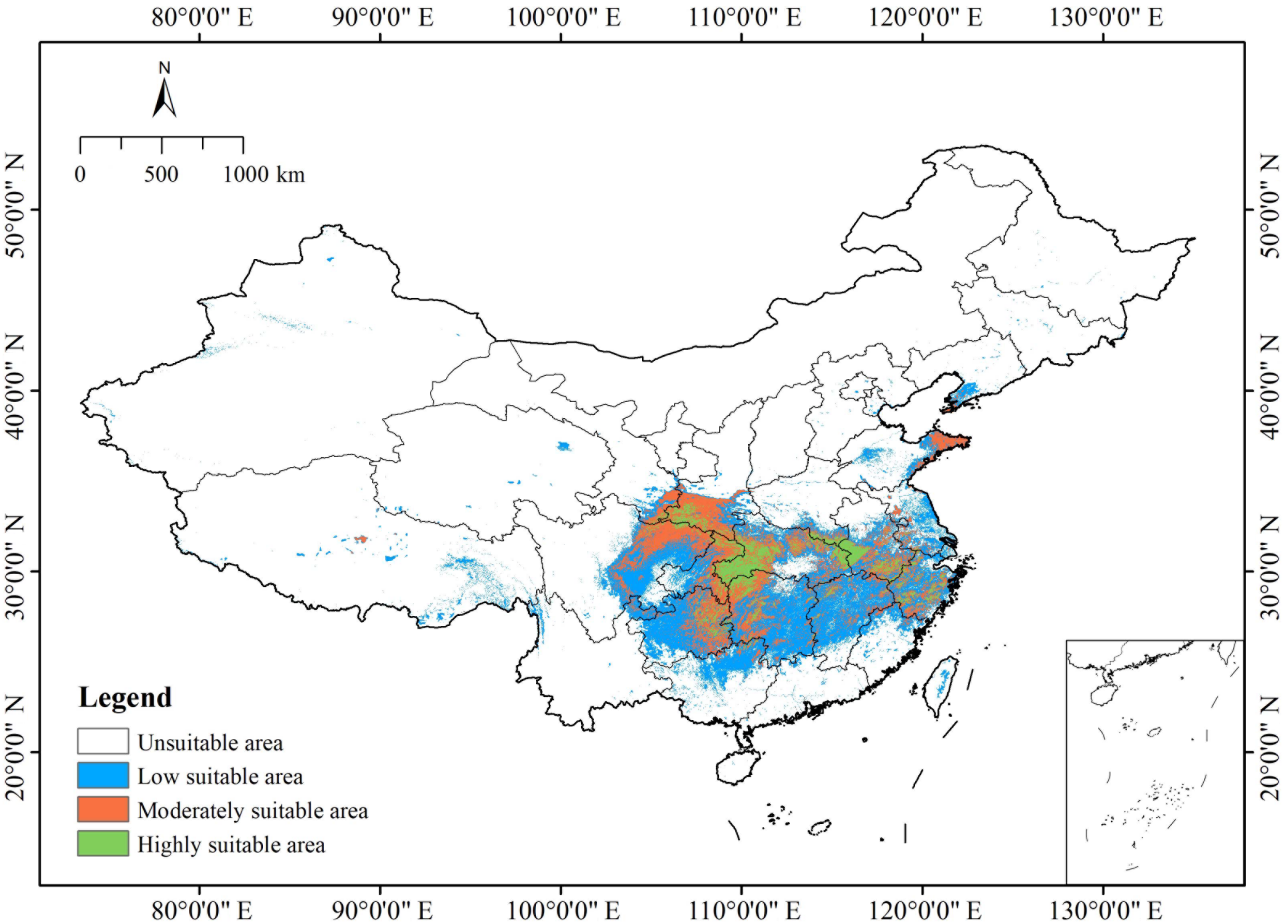
Predicted current distribution of *Changnienia amoena* in China.

The highly suitable habitats for *C. amoena* were mostly located in the western Hubei, the junction area between Shaanxi and Sichuan, eastern Guizhou, northwest Hunan, and the junction between Anhui, Hubei, and Henan provinces, with some areas scattered in Zhejiang and southern Anhui provinces in China. The moderately suitable habitats were primarily on the edges of the highly suitable habitats, including northeast Sichuan, southeast Shaanxi, eastern Chongqing, eastern Guizhou, and eastern Shandong, with few suitable areas scattered in the western Jiangsu, Zhejiang, Hunan, Jiangxi, and Anhui provinces. The low suitable habitats were mainly in Jiangsu, Guizhou, Hunan, Jiangxi, Zhejiang, Anhui, southeastern Sichuan, northern Fujian and Guangxi, the central part of Hubei and Taiwan, and southern Liaoning provinces (Figure 4).

We combined the highly and moderately suitable habitats into the suitable habitat area (Lu, Jiang, and Zhang 2022). For *C. amoena* , the current suitable area for *C. amoena* was 58.33 × 10^4^ km^2^, accounting for 6.08% of China's total territory. The highly suitable area was 14.34 × 10^4^ km^2^, only making up 24.58% of the total suitable area (Table 4), mainly concentrated in southwest Hubei province.

**TABLE 4:** Potential suitable areas of *Changnienia amoena* under different climate scenarios.

Climate scenarios	Low suitable area		Moderately suitable area		Highly suitable area		Suitable area (moderately and highly)	
	Area (×10^4^ km^2^)	Trend (%)	Area (×10^4^ km^2^)	Trend (%)	Area (×10^4^ km^2^)	Trend (%)	Area (×10^4^ km^2^)	Trend (%)
Last interglacial (LIG)	56.79	↓27.70	44.13	↑0.32	20.38	↑42.12	64.51	↑10.59
Mid‐holocene (MH)	49.83	↓36.56	41.89	↓4.77	33.36	↑57.01	75.25	↑29.01
Current	78.55	—	43.99	—	14.34	—	58.33	—
2050s								
RCP2.6	88.09	↑12.14	37.49	↓14.78	12.54	↓12.55	50.03	↓14.23
RCP4.5	86.18	↑9.71	34.18	↓22.30	14.31	↓0.21	48.49	↓16.87
RCP8.5	68.29	↓13.06	44.88	↑2.02	17.35	↑20.99	62.23	↑6.69
2070s								
RCP2.6	69.11	↓12.02	43.90	↓0.20	27.74	↑93.44	71.64	↑22.82
RCP4.5	80.46	↑2.43	37.67	↓14.37	16.92	↑17.99	54.59	↓6.41
RCP8.5	93.90	↑19.54	41.22	↓6.30	13.87	↓3.28	55.09	↓5.55
The mean value of six future climate scenarios	81.01	↑3.13	39.89	↓9.32	17.12	↑19.39	57.01	↓2.26

*Note:* Up arrow (↑) means increase; down arrow (↓) means decrease.

**FIGURE 5:**
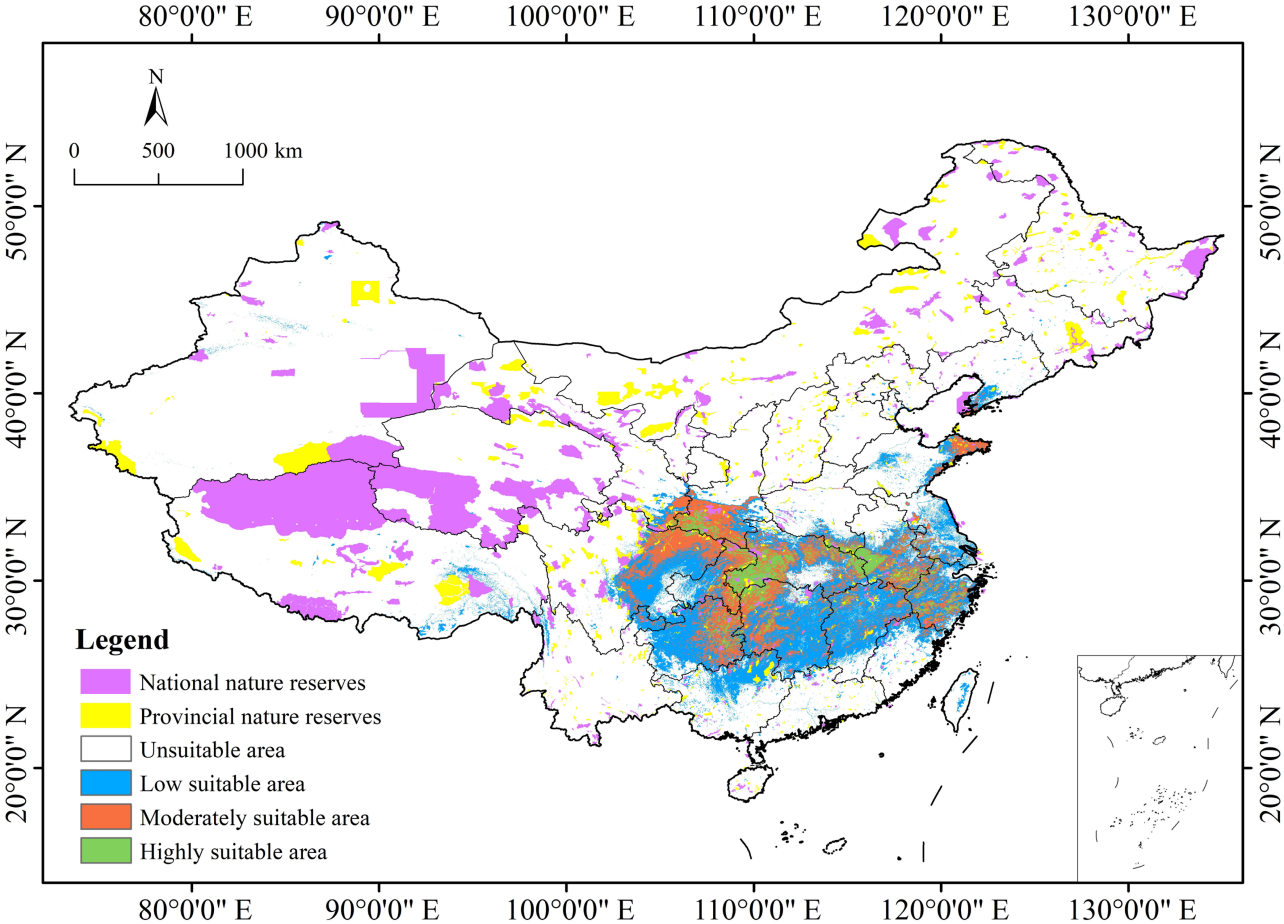
The overlap of the current suitable habitat of *Changnienia amoena* with national and provincial nature reserves in China.

Under current climatic conditions, the suitable habitat of *C. amoena* within national‐level protected area was 2.61 × 10^4^ km^2^. The coverage ratio of the national protected area for this species' suitable habitat was 4.48%, only accounting for 2.69% of the total protected area (i.e., including national and provincial) in China. These areas were mainly located in Shennongjia in Hubei province, Badagong Mountain in Hunan province, Anhui province, and the junction regions of Shaanxi and Sichuan provinces (Figure 5). Likewise, the suitable habitat of *C. amoena* within provincial protected area was 1.95 × 10^4^ km^2^. The coverage ratio of the provincial protected area for this species' suitable habitat was 3.33%, only accounting for 2.01% of the total protected area in China. Therefore, the vast majority of *C. amoena* 's suitable habitat is not effectively protected.

### 3.4 Past and Future Distribution Shift

**FIGURE 6:**
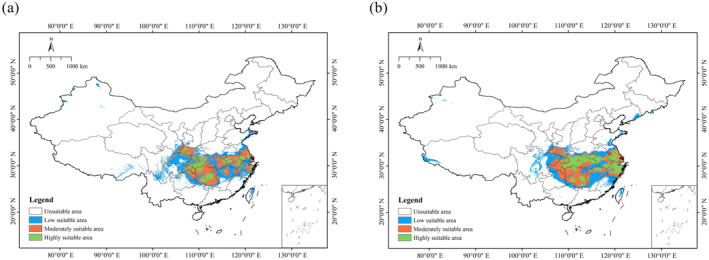
Predicted the Last Inter Glacial (LIG) (a) and the mid‐holocene (MH) (b) distributions of *Changnienia amoena* in China.

In the past, the potential suitable habitat of *C. amoena* was larger than the current one, and it was mainly concentrated in Hubei, Hunan, Jiangxi, Zhejiang, Jiangsu, and Anhui provinces, especially during the MH period (Figure 6). During the LIG period, the suitable area was 64.51 × 10^4^ km^2^, which had an increase of 10.59% compared to the present. Likewise, a significant expansion occurred during the MH period, with the largest suitable area of 75.25 × 10^4^ km^2^, which had an increase of 29.01% relative to the current (Table 4).

**FIGURE 7:**
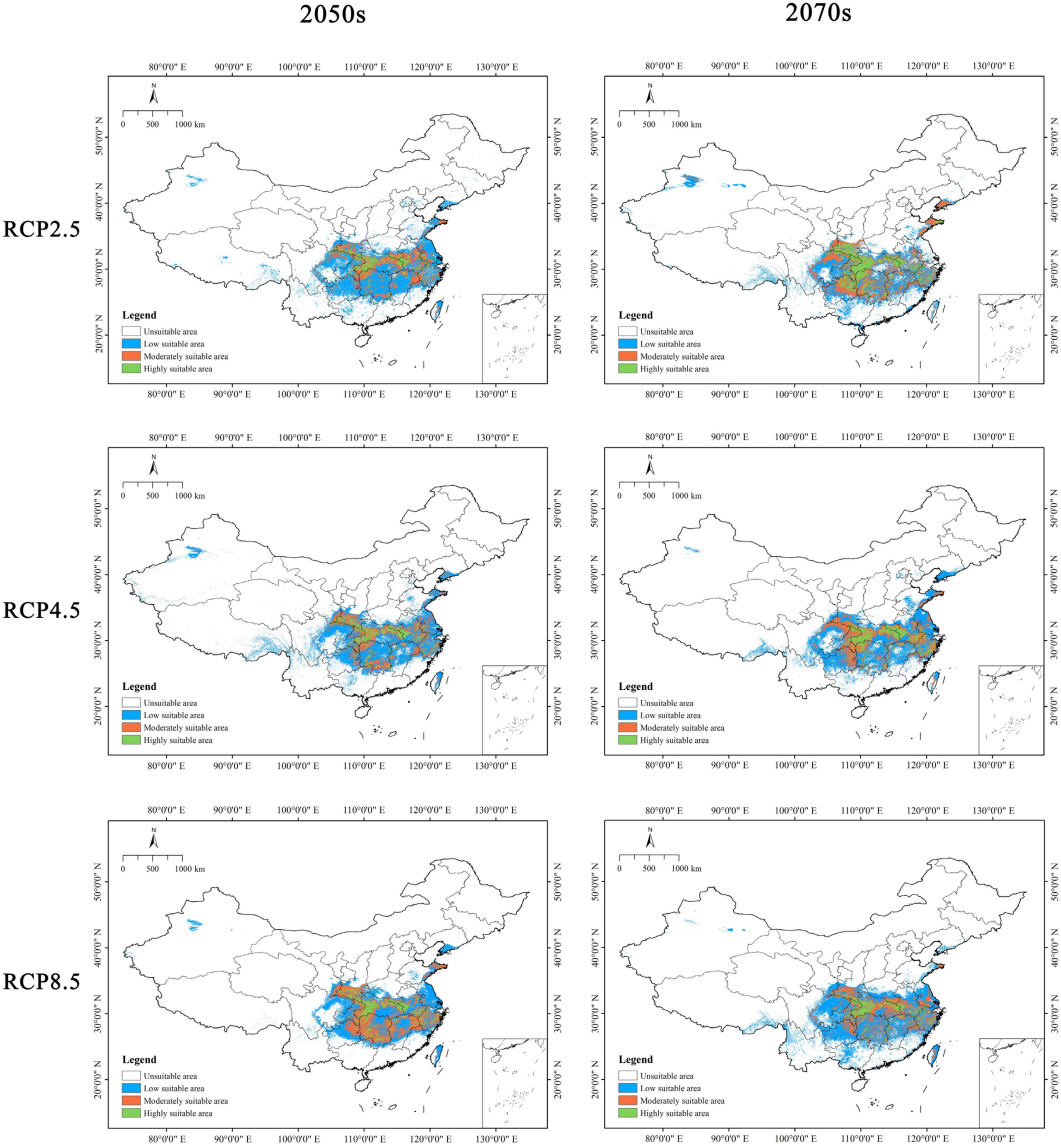
Potential suitable distributions of *Changnienia amoena* in China under different future climatic scenarios (RCP 2.6, RCP 4.5, and RCP 8.5) in the 2050s and 2070s using the ensemble model.

As shown in Figure 7, under six future climate scenarios, the highly suitable area for *C. amoena* was expected to decrease in northeastern Hunan and at the junction between Shaanxi and Sichuan provinces, while it was anticipated to increase in central Hubei and at the junction among Anhui, Hubei, and Henan provinces. The moderately suitable area for *C. amoena* was mainly expected to reduce at the junction between Shaanxi and Sichuan provinces, the junction between Guizhou and Hunan provinces, and in eastern Shandong province (Figure 7).

In the future, the suitable area of *C. amoena* in different periods (2050s and 2070s) would respond distinctively to climate change. Overall, the future mean suitable area for *C. amoena* was predicted to be 57.01 × 10^4^ km^2^, indicating a decreasing trend. It declined by 2.26% compared to the current area. In the 2050s, its suitable habitat decreased under both low and medium emission scenarios (i.e., RCP 2.6 and RCP 4.5), while it increased under high emission scenario (RCP 8.5). Under RCP 4.5, the suitable area was expected to be 48.49 × 10^4^ km^2^, with a maximum decline of 16.87% compared to the current. In the 2070s, its suitable habitat increased under RCP 2.6, while it decreased under RCP 4.5 and RCP 8.5. Under RCP 2.6, the suitable area for *C. amoena* was expected to amount to 71.64 × 10^4^ km^2^, with a largest increase of 22.82% relative to the current. The suitable area for *C. amoena* was predicted to decrease in the 2050s and 2070s under RCP 4.5 (Table 4).

In summary, from the past to the current and subsequently to future climate scenarios, the suitable area change for *C. amoena* showed a fluctuating type. The suitable area decreased in the current and future compared to the two past periods, with severe habitat fragmentation.

### 3.5 Centroid Change of Suitable Area

**FIGURE 8:**
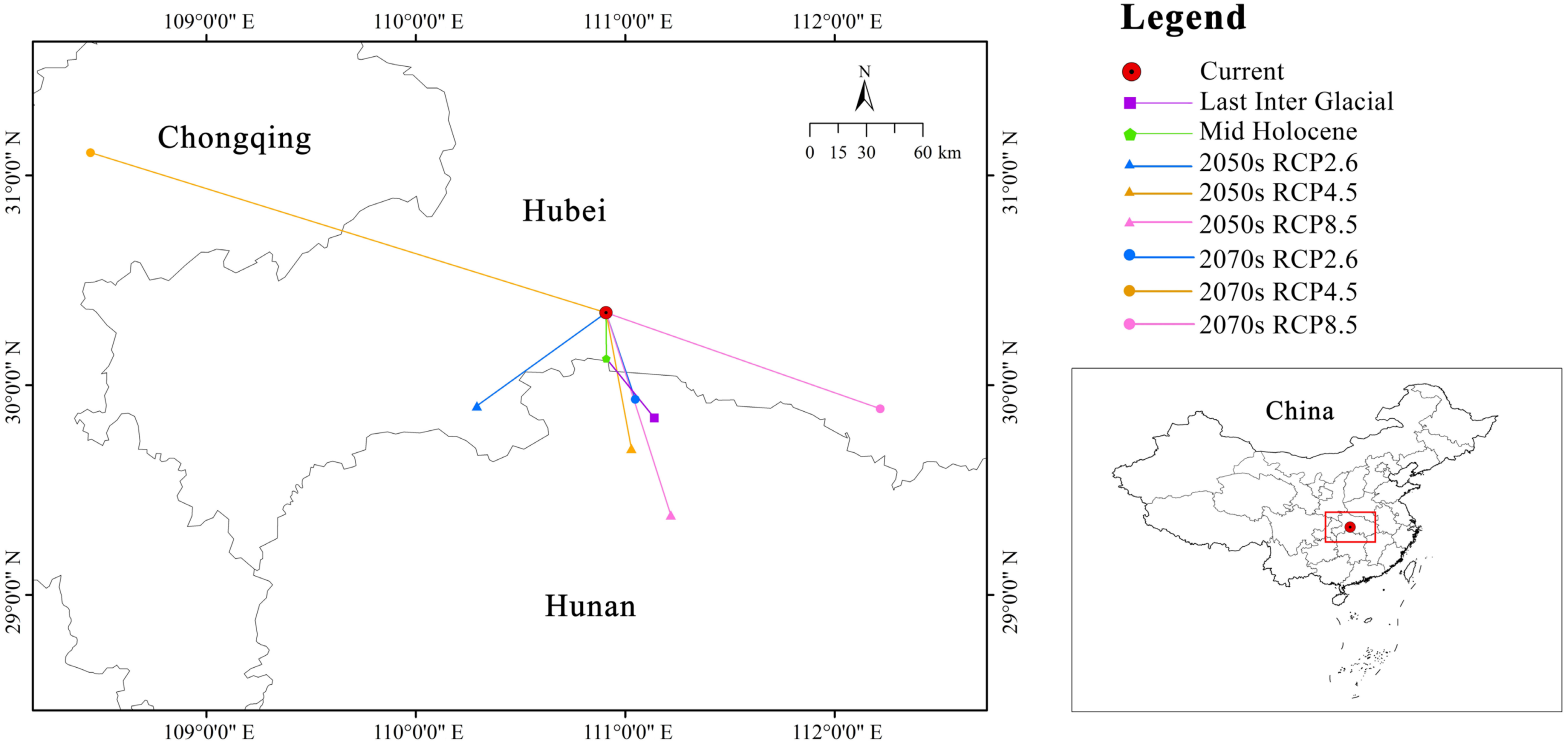
Shifting of the core distribution in suitable areas of *Changnienia amoena* in China.

From the last interglacial to the mid‐holocene, the centroid (111.1392° E, 29.8431° N) of *C. amoena* shifted 38.47 km in a northwestward direction. From the MH to current period, the centroid (110.9099° E, 30.1266° N) shifted 24.30 km towards the north. Under six future climate scenarios, the centroid's (110.9077° E, 30.3452° N) average shifting distance for *C. amoena* was expected to be 19.72 km with the majority of the movements directed to southeastward. Under RCP 2.6 scenario, the centroid shifted southwestward with the distance of 77.18 km in the 2070s, while it shifted 47.84 km to the southeast in the 2050s. Under RCP 4.5, it moved northeastward with the furthest distance of 249.89 km in the 2050s and 72.79 km to the southeast in the 2070s. Under RCP 8.5, the centroid shifted 135.78 km southeastward in the 2050s and 111.22 km to the southeast in the 2070s (Figure 8). Moreover, the past and future centroid shifts of *C. amoena* were expected in the junction area of Hubei, Hunan, and Chongqing provinces.

Collectively, the centroid shifting pattern of *C. amoena* was circuitous, initially shifting northwestward from the LIG to the MH, and subsequently northward towards the current. From the current to future, the centroid is generally expected to shift southeastward.

## 4 Discussion

### 4.1 Model Selection and Evaluation

Recent studies have shown that the accuracy of species distribution predictions can be considerably improved by applying ensemble model rather than a single model (Stewart et al. 2022; Valavi et al. 2023; Dong et al. 2024). For example, Xian et al. (2022) used an ensemble model to predict the distribution pattern of *Ageratina adenophora* in China under climate change. Their results show that AUC and TSS values for the ensemble model are 0.993 and 0.925, respectively, indicating that the ensemble model performed well in terms of predictive accuracy. In this study, we first employed the Biomod2, which comprised ten individual models, to predict the potential geographical distribution of endangered *C. amoena* under different climate scenarios in China. Then, we selected three of them (RF, MaxEnt, and GBM) in terms of AUC and TSS values. Finally, we developed an ensemble model whose AUC and TSS are > 0.9, respectively. This indicates that such an ensemble model presents a superior predictive performance relative to each of the ten individual models (Table 2). Furthermore, all known occurrence points of *C. amoena* fall within the forecasted distribution range under the current climate scenario (Figure 4). This further suggests that the prediction outcomes of the ensemble model used in this study are reliable. Therefore, we used the ensemble model to predict the potential distribution under past (i.e., LIG and MH), current, and six future climate scenarios, respectively.

### 4.2 Key Influencing Factors of *C. amoena*

In this study, we established an ensemble model to determine the optium suitability of *C. amoena* in terms of different environmental factors (i.e., climate, soil, and topography). Our results have shown that the main factors influencing its potential distribution are mean diurnal range (Bio2), minimum temperature of the coldest month (Bio6), temperature seasonality (Bio4), and precipitation of the warmest quarter (Bio18), and that the sum of their percent contribution is up to 86.2% (Table 3). Hence, this indicates that bioclimate is more crucial factor affecting the potential distribution of *C. amoena* than soil and topography in China. Liu et al. (2010) contends that wild Orchids are exquisitely sensitive to alterations in temperature and moisture. *C. amoena* is currently distributed in more than 13 provinces in China, covering a wide range and spanning subtropical and warm temperate zones. Among the 19 bioclimatic factors, the top three are temperature‐related (i.e., Bio2, Bio6, and Bio4), accounting for 72.9% of the total percent contribution for *C. amoena* . Especially, for *C. amoena,* Bio2 contributes the maximum, taking up 43.7%, which is in accordance with endangered orchid *Cremastra appendiculata* (Li et al. 2022). This indicates that temperature‐related variables may play a more important role in limiting the potential distribution of *C. amoena* than precipitation‐related variables.

In other words, water plays a much smaller part in limiting the distribution of *C. amoena* relative to temperature. This may be associated with such a structure, namely, pseudobulb. Most Orchids, including *C. amoena* (Figure 1c), have apparent pseudobulbs. Pseudobulb is able to hold water, store nutrients, and perform photosynthesis in Orchidaceae, and such a bulblike enlargement of the stem can help orchids for survival and growth under adverse environmental conditions (Ng and Hew 2000; Li and Zhang 2019). In addition, *C. amoena* is a shade‐tolerant herb, and it prefers to grow in soils with much rich humus (Wu and Raven 2009). Therefore, we think that such a structure may be one of the major reasons for affecting its distribution besides bioclimatic factors.

In brief, we have identified for the first time the key environmental factors affecting the distribution of *C. amoena* and further determined their corresponding optimal ranges. Namely, *C. amoena* grows well in humid habitats with low variation in mean diurnal range and temperature seasonality.

### 4.3 Current Suitable Area of *C. amoena*

The predicted outcome from the Biomod2 shows that the potential suitable area for *C. amoena* in China is 58.33 × 10^4^ km^2^ under the current climate scenario, accounting for only 6.08% of China's total territory (Table 4). The area is mainly distributed in Anhui, Guizhou, Henan, Hubei, Hunan, Shaanxi, and Sichuan provinces (Figure 4). The current suitable range of *C. amoena* involves 18 provinces in China, which is clearly more than eight provinces recorded in *Flora of China*. Therefore, the actual distribution range of *C. amoena* , rather than its actual occupied area in China, is much larger than previously known, and it is also evidently greater than the predicted outcome (2.44 × 10^4^ km^2^) within Jiangxi Province (Chen 2019).

Furthermore, our predictive results also indicate that such provinces as Chongqing, Guangxi, Guizhou, Gansu, and Henan, which are not recorded in *Flora of China* (Wu and Raven 2009), may indeed have a potential distribution of *C. amoena* in China. An example in point is Guizhou province, which has both moderately and highly suitable areas (Figure 4). Zhang and Yang (2010) reported the distribution of *C. amoena* under an evergreen broad‐leaved forest at Fanjingshan National Nature Reserve, northeastern Guizhou Province. At the same time, we tend to think that some provinces like Fujian probably have wild populations of *C. amoena*, although there has been no record in these areas so far.

In fact, there is an obvious habitat fragmentation for the majority of provinces with the suitability of *C. amoena* in China. This is consistent with the discontinuous distribution pattern of *C. amoena* in the five sampled provinces (Li and Ge 2006). This orchid usually has only one flower per individual (Figure 1d). It does not secrete nectar and provides no reward for pollinators, thus falling into a deceptive pollination mode (Sun et al. 2006). Moreover, only one species of bumblebees, namely *Bombus trifasciatus* , is considered as its legitimate pollinator among the 15 candidate pollinating insects (Sun, Luo, and Ge 2003). All these may result in its low fruit set rate.

Therefore, we believe that climate change and biological characteristics are jointly responsible for the habitat fragmentation of *C. amoena* under the current climate scenario.

### 4.4 Suitable Area Change Under Different Climate Scenarios

According to our analysis, the suitable habitat area for *C. amoena* during the LIG period was 64.51 × 10^4^ km^2^, and it would expand to 75.25 × 10^4^ km^2^ during the mid‐holocene. Compared to the current period, its suitable area increased by 10.59% and 29.01% during LIG and MH, respectively (Table 4). Therefore, the suitable areas in the past were significantly larger than the current one. The climate during the LIG period was warm and dry with less precipitation (Yan et al. 2022), which could limit the growth of *C. amoena* . The mid‐holocene was the last major warm period, characterized by a warm and moist climate with more precipitation (He et al. 2022). The overall warm and humid climatic conditions during this period may be more conducive to the growth and reproduction of *C. amoena* , resulting in a significant increase and concentration of its suitable area.

Conversely, under six future climate scenarios, the average suitable habitat area for *C. amoena* is 57.01 × 10^4^ km^2^, decreased by 2.26% of the current distribution (Table 4). In different periods, climate change has different effects on its suitable areas. The average suitable area is 53.58 × 10^4^ km^2^ in the 2050s and 60.44 × 10^4^ km^2^ in the 2070s, respectively. Under different emission conditions, there is a slight fluctuation in the suitable area of *C. amoena* . Under the RCP2.6 climate scenario, the suitable area for *C. amoena* is expected to decrease in 2050s but increase in 2070s. Under the RCP8.5 climate scenario, its suitable area is expected to increase in 2050s but decrease in 2070s. In contrast, under the RCP4.5 climate scenario, its suitable area will decrease both in 2050s and 2070s. Collectively, except 2050s‐RCP8.5 and 2070s‐RCP2.6, there is a moderate decreasing trend for each of the other four future scenarios in the suitable area.

Overall, *C. amoena* shows a gradual decrease in the suitable area from the past to present and future, with intensifying habitat fragmentation.

Under future climate scenarios, the centroid of *C. amoena* largely shows a southeastward shifting (Figure 8). This is consistent with the shifting directions of *Lilium polyphyllum* (Dhyani et al. 2021) and *Osmanthus fragrans* (Kong et al. 2021) in China in the future. Therefore, the shifting of *C. amoena* towards low latitude in China may be related to its preference for growing in warm and humid regions.

### 4.5 Conservation Implications for *C. amoena*

Our model projects that the current suitable area of 58.33 × 10^4^ km^2^ for *C. amoena* is significantly larger than the known range, across more than 10 provinces in China. Moreover, its suitable area will slightly decrease under future scenarios. At present, *C. amoena* is largely concentrated in Anhui, Chongqing, Hubei, Hunan, and other provinces where there are complex and diverse climatic conditions and vegetation types. Accordingly, it is most likely to have its wild populations in these regions. Therefore, we recommend conducting supplemental survey concerning *C. amoena* wild populations in China, including the whole Hubei, northern Hunan, southeastern Jiangsu, northern Jiangxi, and southern Shaanxi, especially in northern Fujian where no *C. amoena* population has been reported so far.

In this study, by overlaying the potential distribution area of *C. amoena* with national and provincial nature reserves, we find that only 4.48% of the suitable area for *C. amoena* is located within national nature reserves and 3.33% within provincial nature reserves, respectively. This indicates that over 90% of the suitable habitat for *C. amoena* is in a zero‐protection status, which may be a primary reason for the “endangered” category of wild *C. amoena* . This orchid is a national second‐grade protected plant species. Nature reserves are the most effective way for in situ conservation of wild orchids (Jin 2012; Qin et al. 2012; Zhang, Du, et al. 2015; Zhang, Yan, et al. 2015). Therefore, we propose to enlarge the nature reserve around which *C. amoena* is relatively concentrated in distribution or to establish plant natural mini‐reserves/protected sites.

In view of its high ornamental and medicinal value, together with the adverse effect of climate change on its distribution, we recommend selecting appropriate sites for *C. amoena* introduction and cultivation in light of its projected suitable habitats.

## 5 Conclusions

We first developed an ensemble model comprising three individual models (i.e., RF, MaxEnt, and GBM) from the Biomod2 in this study. Then, we combined occurrence records of the endangered and endemic *C. amoena* in China with climate, terrain, and soil variables to successfully project its potential distribution under various climate scenarios. Our results show that mean diurnal range (Bio2), minimum temperature of the coldest month (Bio6), temperature seasonality (Bio4), and precipitation of the warmest quarter (Bio18) are the key environmental factors limiting its geographical distribution. For the first time, we have determined its current suitable area of 58.33 × 10^4^ km^2^, accounting for only 6.08% of China's total territory. The orchid is mainly distributed in central and eastern China, with the larger range than known. Its suitable area is considerably larger during the LIG and MH periods than currently. Under future climate scenarios, its suitable area will averagely decrease by 2.26%. Our findings reveal a declining trend in the suitable area of *C. amoena* from the past to present and future. This demonstrates that climate change has an adverse impact on this orchid, with increasing habitat fragmentation. Our analysis further shows that over 90% of the suitable area of *C. amoena* is outside national and provincial nature reserves in China, suggesting significant conservation gaps. Therefore, we recommend expanding protected areas or establishing new conservation sites for *C. amoena* . Furthermore, our study can also provide reference for conservation and management for other endangered orchids in China under climate change. In addition, only environmental factors are used in the present study, and other factors such as human disturbance and land use change can be taken into account in the future.

## Author Contributions

**Ting Liu:** data curation (equal), formal analysis (equal), writing – original draft (equal). **Hanwei Cai:** data curation (equal), investigation (equal). **Guangfu Zhang:** conceptualization (equal), investigation (equal), writing – review and editing (lead).

## Conflicts of Interest

The authors declare no conflicts of interest.

## Supporting information


**Table S1.** Latitude and longitude coordinates of 93 occurrence records of the endangered *Changnienia amoena* in China.

## Data Availability

The original contributions presented in the study are included in the article/Supporting Information. Further inquiries can be directed to the corresponding author.
