# Supplementary material for: Assessment of Climate Change Impacts on the Distribution of Endangered and Endemic Changnienia amoena (Orchidaceae) Using Ensemble Modeling and Gap Analysis in China
Source: Ecol Evol. 2024 Nov 25;14(11):e70636. doi: 10.1002/ece3.70636 (PMC11588355; doi:10.1002/ece3.70636)
Supplement: Supplementary file 1 — Table S1. Latitude and longitude coordinates of 93 occurrence records of the endangered Changnienia amoena in China. [file ECE3-14-e70636-s001.docx]

**Appendix**

**Table S1 Latitude and longitude coordinates of 93 occurrence records of the endangered *Changnienia amoena* in China.**

| **Species** | **Longitude (°)** | **Latitude (°)** |
| --- | --- | --- |
| *Changnienia amoena* | 116.02 | 31.19 |
| *Changnienia amoena* | 117.50 | 30.10 |
| *Changnienia amoena* | 116.31 | 31.36 |
| *Changnienia amoena* | 115.92 | 31.69 |
| *Changnienia amoena* | 105.74 | 33.03 |
| *Changnienia amoena* | 105.50 | 32.94 |
| *Changnienia amoena* | 108.70 | 27.92 |
| *Changnienia amoena* | 114.04 | 32.13 |
| *Changnienia amoena* | 110.01 | 30.45 |
| *Changnienia amoena* | 109.82 | 30.19 |
| *Changnienia amoena* | 110.37 | 31.44 |
| *Changnienia amoena* | 115.91 | 31.01 |
| *Changnienia amoena* | 114.61 | 31.30 |
| *Changnienia amoena* | 114.66 | 31.47 |
| *Changnienia amoena* | 116.01 | 31.00 |
| *Changnienia amoena* | 115.68 | 30.75 |
| *Changnienia amoena* | 115.81 | 31.10 |
| *Changnienia amoena* | 110.27 | 31.47 |
| *Changnienia amoena* | 110.10 | 31.46 |
| *Changnienia amoena* | 110.54 | 30.74 |
| *Changnienia amoena* | 109.86 | 29.00 |
| *Changnienia amoena* | 110.17 | 29.42 |
| *Changnienia amoena* | 110.54 | 29.13 |
| *Changnienia amoena* | 119.09 | 32.14 |
| *Changnienia amoena* | 118.91 | 30.11 |
| *Changnienia amoena* | 119.44 | 30.37 |
| *Changnienia amoena* | 109.88 | 31.08 |
| *Changnienia amoena* | 104.05 | 30.65 |
| *Changnienia amoena* | 110.39 | 31.44 |
| *Changnienia amoena* | 116.04 | 31.41 |
| *Changnienia amoena* | 119.46 | 30.34 |
| *Changnienia amoena* | 110.08 | 31.29 |
| *Changnienia amoena* | 118.20 | 29.38 |
| *Changnienia amoena* | 114.63 | 31.54 |
| *Changnienia amoena* | 104.82 | 31.99 |
| *Changnienia amoena* | 116.03 | 30.99 |
| *Changnienia amoena* | 105.49 | 33.00 |
| *Changnienia amoena* | 109.45 | 30.28 |
| *Changnienia amoena* | 110.98 | 30.83 |
| *Changnienia amoena* | 111.33 | 30.76 |
| *Changnienia amoena* | 113.43 | 32.38 |
| *Changnienia amoena* | 101.50 | 27.42 |
| *Changnienia amoena* | 109.87 | 31.11 |
| *Changnienia amoena* | 109.36 | 27.57 |
| *Changnienia amoena* | 113.01 | 28.21 |
| *Changnienia amoena* | 118.80 | 32.06 |
| *Changnienia amoena* | 112.73 | 27.24 |
| *Changnienia amoena* | 115.97 | 29.34 |
| *Changnienia amoena* | 115.99 | 29.67 |
| *Changnienia amoena* | 119.17 | 31.92 |
| *Changnienia amoena* | 119.44 | 30.35 |
| *Changnienia amoena* | 110.26 | 26.49 |
| *Changnienia amoena* | 116.00 | 29.59 |
| *Changnienia amoena* | 110.08 | 31.22 |
| *Changnienia amoena* | 109.00 | 29.60 |
| *Changnienia amoena* | 115.92 | 29.49 |
| *Changnienia amoena* | 115.80 | 31.14 |
| *Changnienia amoena* | 115.78 | 31.17 |
| *Changnienia amoena* | 107.09 | 29.18 |
| *Changnienia amoena* | 119.32 | 31.79 |
| *Changnienia amoena* | 115.96 | 29.46 |
| *Changnienia amoena* | 103.95 | 31.84 |
| *Changnienia amoena* | 116.02 | 29.52 |
| *Changnienia amoena* | 115.93 | 29.52 |
| *Changnienia amoena* | 114.08 | 31.82 |
| *Changnienia amoena* | 110.68 | 31.75 |
| *Changnienia amoena* | 109.50 | 29.49 |
| *Changnienia amoena* | 110.45 | 34.46 |
| *Changnienia amoena* | 110.61 | 29.05 |
| *Changnienia amoena* | 110.87 | 26.41 |
| *Changnienia amoena* | 111.11 | 26.34 |
| *Changnienia amoena* | 116.58 | 31.09 |
| *Changnienia amoena* | 105.23 | 32.68 |
| *Changnienia amoena* | 110.47 | 25.94 |
| *Changnienia amoena* | 108.62 | 27.55 |
| *Changnienia amoena* | 108.87 | 27.77 |
| *Changnienia amoena* | 114.07 | 31.82 |
| *Changnienia amoena* | 111.93 | 33.51 |
| *Changnienia amoena* | 110.50 | 31.33 |
| *Changnienia amoena* | 110.89 | 31.60 |
| *Changnienia amoena* | 110.03 | 31.21 |
| *Changnienia amoena* | 115.86 | 31.10 |
| *Changnienia amoena* | 115.85 | 30.98 |
| *Changnienia amoena* | 110.72 | 26.51 |
| *Changnienia amoena* | 111.13 | 26.71 |
| *Changnienia amoena* | 109.36 | 32.03 |
| *Changnienia amoena* | 106.46 | 32.82 |
| *Changnienia amoena* | 106.26 | 32.83 |
| *Changnienia amoena* | 106.01 | 33.03 |
| *Changnienia amoena* | 109.53 | 31.95 |
| *Changnienia amoena* | 108.36 | 31.95 |
| *Changnienia amoena* | 114.92 | 28.87 |
| *Changnienia amoena* | 114.15 | 26.64 |
| *Changnienia amoena* | 116.02 | 31.19 |
| *Changnienia amoena* | 117.50 | 30.10 |
| *Changnienia amoena* | 116.31 | 31.36 |
| *Changnienia amoena* | 115.92 | 31.69 |
| *Changnienia amoena* | 105.74 | 33.03 |
| *Changnienia amoena* | 105.50 | 32.94 |
| *Changnienia amoena* | 108.70 | 27.92 |
| *Changnienia amoena* | 114.04 | 32.13 |
| *Changnienia amoena* | 110.01 | 30.45 |
| *Changnienia amoena* | 109.82 | 30.19 |
| *Changnienia amoena* | 110.37 | 31.44 |
| *Changnienia amoena* | 115.91 | 31.01 |
| *Changnienia amoena* | 114.61 | 31.30 |
| *Changnienia amoena* | 114.66 | 31.47 |
| *Changnienia amoena* | 116.01 | 31.00 |
| *Changnienia amoena* | 115.68 | 30.75 |
| *Changnienia amoena* | 115.81 | 31.10 |
| *Changnienia amoena* | 110.27 | 31.47 |
| *Changnienia amoena* | 110.10 | 31.46 |
| *Changnienia amoena* | 110.54 | 30.74 |
| *Changnienia amoena* | 109.86 | 29.00 |
| *Changnienia amoena* | 110.17 | 29.42 |
| *Changnienia amoena* | 110.54 | 29.13 |
| *Changnienia amoena* | 119.09 | 32.14 |
| *Changnienia amoena* | 118.91 | 30.11 |
| *Changnienia amoena* | 119.44 | 30.37 |
| *Changnienia amoena* | 109.88 | 31.08 |
| *Changnienia amoena* | 104.05 | 30.65 |
| *Changnienia amoena* | 110.39 | 31.44 |
| *Changnienia amoena* | 116.04 | 31.41 |
| *Changnienia amoena* | 119.46 | 30.34 |
| *Changnienia amoena* | 110.08 | 31.29 |
| *Changnienia amoena* | 118.20 | 29.38 |
| *Changnienia amoena* | 114.63 | 31.54 |
| *Changnienia amoena* | 104.82 | 31.99 |
| *Changnienia amoena* | 116.03 | 30.99 |
| *Changnienia amoena* | 105.49 | 33.00 |
| *Changnienia amoena* | 109.45 | 30.28 |
| *Changnienia amoena* | 110.98 | 30.83 |
| *Changnienia amoena* | 111.33 | 30.76 |
| *Changnienia amoena* | 113.43 | 32.38 |
| *Changnienia amoena* | 101.50 | 27.42 |
| *Changnienia amoena* | 109.87 | 31.11 |
| *Changnienia amoena* | 109.36 | 27.57 |
| *Changnienia amoena* | 113.01 | 28.21 |
| *Changnienia amoena* | 118.80 | 32.06 |
| *Changnienia amoena* | 112.73 | 27.24 |
| *Changnienia amoena* | 115.97 | 29.34 |
| *Changnienia amoena* | 115.99 | 29.67 |
| *Changnienia amoena* | 119.17 | 31.92 |
| *Changnienia amoena* | 119.44 | 30.35 |
| *Changnienia amoena* | 110.26 | 26.49 |
| *Changnienia amoena* | 116.00 | 29.59 |
| *Changnienia amoena* | 110.08 | 31.22 |
| *Changnienia amoena* | 109.00 | 29.60 |
| *Changnienia amoena* | 115.92 | 29.49 |
| *Changnienia amoena* | 115.80 | 31.14 |
| *Changnienia amoena* | 115.78 | 31.17 |
| *Changnienia amoena* | 107.09 | 29.18 |
| *Changnienia amoena* | 119.32 | 31.79 |
| *Changnienia amoena* | 115.96 | 29.46 |
| *Changnienia amoena* | 103.95 | 31.84 |
| *Changnienia amoena* | 116.02 | 29.52 |
| *Changnienia amoena* | 115.93 | 29.52 |
| *Changnienia amoena* | 114.08 | 31.82 |
| *Changnienia amoena* | 110.68 | 31.75 |
| *Changnienia amoena* | 109.50 | 29.49 |
| *Changnienia amoena* | 110.45 | 34.46 |
| *Changnienia amoena* | 110.61 | 29.05 |
| *Changnienia amoena* | 110.87 | 26.41 |
| *Changnienia amoena* | 111.11 | 26.34 |
| *Changnienia amoena* | 116.58 | 31.09 |
| *Changnienia amoena* | 105.23 | 32.68 |
| *Changnienia amoena* | 110.47 | 25.94 |
| *Changnienia amoena* | 108.62 | 27.55 |
| *Changnienia amoena* | 108.87 | 27.77 |
| *Changnienia amoena* | 114.07 | 31.82 |
| *Changnienia amoena* | 111.93 | 33.51 |
| *Changnienia amoena* | 110.50 | 31.33 |
| *Changnienia amoena* | 110.89 | 31.60 |
| *Changnienia amoena* | 110.03 | 31.21 |
| *Changnienia amoena* | 115.86 | 31.10 |
| *Changnienia amoena* | 115.85 | 30.98 |
| *Changnienia amoena* | 110.72 | 26.51 |
| *Changnienia amoena* | 111.13 | 26.71 |
| *Changnienia amoena* | 109.36 | 32.03 |
| *Changnienia amoena* | 106.46 | 32.82 |
| *Changnienia amoena* | 106.26 | 32.83 |
| *Changnienia amoena* | 106.01 | 33.03 |
| *Changnienia amoena* | 109.53 | 31.95 |
| *Changnienia amoena* | 108.36 | 31.95 |
| *Changnienia amoena* | 114.92 | 28.87 |
| *Changnienia amoena* | 114.15 | 26.64 |
